# A Multi-Layered Origami Tactile Sensory Ring for Wearable Biomechanical Monitoring

**DOI:** 10.3390/bios15010008

**Published:** 2024-12-27

**Authors:** Rajat Subhra Karmakar, Hsin-Fu Lin, Jhih-Fong Huang, Jui-I Chao, Ying-Chih Liao, Yen-Wen Lu

**Affiliations:** 1 Department of Biomechatronics Engineering, National Taiwan University, Taipei 10617, Taiwan; rjtkarmakar@gmail.com; 2 Master Program of Sports Facility Management and Health Promotion, National Taiwan University, Taipei 10617, Taiwan; hsinfu@ntu.edu.tw; 3 Department of Biological Science and Technology, National Yang Ming Chiao Tung University, Hsinchu 30010, Taiwan; pantinz.jr@gmail.com (J.-F.H.); jichao@nycu.edu.tw (J.-I.C.); 4 Department of Chemical Engineering, National Taiwan University, Taipei 10617, Taiwan; liaoy@ntu.edu.tw; 5 Institute of Biotechnology, National Taiwan University, Taipei 10617, Taiwan

**Keywords:** electrical contact resistance, flexible origami tactile sensor, origami ring, conductive composite ink, grip strength, pulse transit time

## Abstract

An origami-based tactile sensory ring utilizing multilayered conductive paper substrates presents an innovative approach to wearable health applications. By harnessing paper’s flexibility and employing origami folding, the sensors integrate structural stability and self-packaging without added encapsulation layers. Knot-shaped designs create loop-based systems that secure conductive paper strips and protect sensing layers. Demonstrating a sensitivity of 3.8 kPa^−1^ at subtle pressures (0–0.05 kPa), the sensors detect both minimal stimuli and high-pressure inputs. Electrical modeling of various origami configurations identifies designs with optimized performance with a pentagon knot offering higher sensitivity to support high-sensitivity needs. Meanwhile a square knot provides greater precision and quicker recovery, balancing sensitivity and stability for real-time feedback devices. The enhanced elastic modulus from folds remains within human skin’s elasticity range, ensuring comfort. Applications include grip strength monitoring and pulse rate detection from the thumb, capturing pulse transit time (PTT), an essential cardiovascular biomarker. This design shows the potential of origami-based tactile sensors in creating versatile, cost-effective wearable health monitoring systems.

## 1. Introduction

The increasing emphasis on physical health, along with advancements in wearable technology and flexible electronics, has led to a surging demand for structured, data-driven fitness programs [1,2,3]. Wearable devices, in particular, enable individuals to monitor their health and fitness metrics with unprecedented precision, enhancing both performance and overall well-being [4,5,6,7,8]. One significant development is the advancement of tactile sensors, which are valued for their flexibility, lightweight design, and ability to detect a broad range of pressures—from subtle forces like pulse detection (0–1 kPa) to higher pressures such as grip strength measurement (>20 kPa)—making them invaluable for monitoring physiological signals during physical activity [9,10,11,12].

To meet the demand for flexible and sensitive tactile sensors, polymer-based composites have emerged as promising sensing materials [13,14,15,16]. These composites combine the inherent elasticity and flexibility of polymers with the conductive properties of fillers such as graphene, carbon nanotubes, reduced graphene oxide, and carbon black [14,15,16,17,18,19,20,21]. By creating a percolated network within the polymer matrix, they respond to mechanical stimuli by converting them into electrical signals [17,18,19]. One particularly advantageous feature of polymer-based composites is their unique interfacial properties when two polymer-coated surfaces come into contact, which is highly beneficial for pressure sensing based on changes in electrical contact resistance (ECR) [15,22,23,24,25,26,27,28]. Unlike rigid metal coatings, polymer surfaces deform under applied pressure, increasing the true contact area more effectively and leading to more pronounced changes in resistance [15,23,24,25,26,27,29]. This enhanced sensitivity to pressure variations, along with the ability to accommodate surface roughness and irregularities, makes polymer-based composites especially suitable for ECR-based tactile sensors.

Moreover, ECR-based tactile sensors made of flexible substrates such as polymers, elastomers, and paper have demonstrated enhanced performance [15,23,28]. The combination of flexible substrates with polymer-based composites results in sensors that are lightweight, durable, and capable of maintaining consistent performance under various mechanical stresses [15]. Among these substrates, paper stands out due to its biodegradability, porosity, and cost-effectiveness [21,30,31,32,33]. The fibrous structure of paper facilitates the formation of conductive networks when combined with conductive fillers, and its ability to deform under pressure enhances the ECR sensing mechanism. Paper’s inherent flexibility and foldability offer greater freedom in shaping and deformation than conventional sheet structures, making it an ideal material for developing compact, flexible sensing systems [34,35]. Origami folding—transforming flat sheets into complex shapes without cutting or gluing—enables intricate designs that enhance both structural stability and surface functionality [36,37,38,39,40,41,42,43]. Although studies have explored origami-inspired sensors, few are utilized to simultaneously improve structural flexibility, increase contact area, and incorporate functional packaging within a single, compact form. By investigating the effects of different folding patterns on sensor performance, this study explores how geometric configurations influence ECR variation and optimize sensor properties for targeted pressure ranges and specific applications.

To create an effective tactile sensing system, three origami structures—Love-knot, Square-knot, and Pentagon-knot—were selected for their simplicity, easy-assembly, and adaptability in ECR-based tactile sensors. Constructed with a polyester-graphene composite ink to ensure reliable conductivity, each configuration was modeled with circuit representations to analyze and optimize ECR changes at the folded surfaces. The optimized origami design was incorporated into a wearable ring, then tested for applications including grip strength assessment for physiotherapy and fingertip pulse detection. These tests showed the origami tactile sensor’s strong potential for precise physiological monitoring and health assessments, highlighting how origami-inspired designs elevate sensor performance in practical wearable applications.

## 2. Materials and Methods

### 2.1. Device Fabrication and Assembly

**Figure 1:**
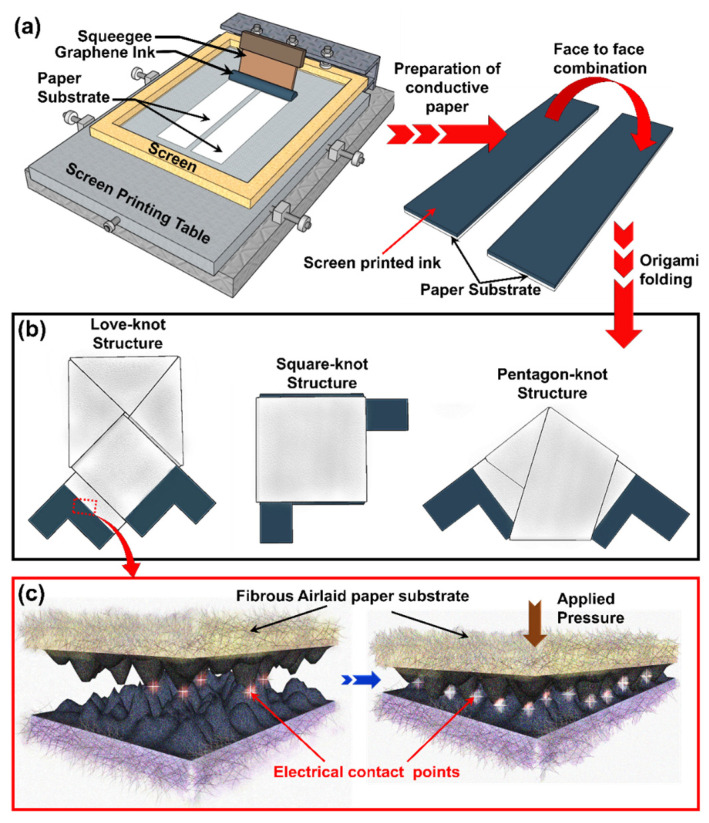
Origami tactile sensors with three different structures and their conduction mechanism: (**a**) Preparation of conductive paper by Screen printing process. (**b**) Schematic diagram of Love-knot, Square-knot, and Pentagon-knot origami structure. Airlaid paper was used as substrate with the graphene ink used as sensing material. (**c**) ECR variation mechanism for tactile sensing.

A 220-µm-thick airlaid paper was selected as the substrate due to its lightweight, flexible, and fibrous properties, and was functionalized with graphene inks to create conductive layers. A graphene-based, water-insoluble composite conductive ink with polyester binder (Graphene Ink, Euflex Corp., New Taipei City, Taiwan) was chosen for its excellent electrical properties and compatibility with screen-printing. This method allows for scalable, low-cost production while maintaining high sensitivity and flexibility, essential for wearable applications. Conductive paper was fabricated by screen-printing the graphene ink onto the airlaid paper using a blank screen with a mesh count of 150 as shown in Figure 1a. The ink was pressed through the mesh with a squeegee to form a uniform conductive layer, followed by baking at 100 °C for 30 min to remove solvent residues and to stabilize the conductive layer without compromising flexibility. The porous and fibrous structure of airlaid paper worked a perfect inert matrix, which was then infused by the graphene ink as the conductive filler. This turned the inert airlaid paper into a conductive structural composite material ideal for tactile-sensing applications.

The conductive paper was cut into 1 cm-wide strips and combined face-to-face to create the primary interfacial resistive layer. Two conductive layers were assembled face-to-face and folded following previously established techniques for Love-knot and Pentagon-knot structures [44], while a novel folding method was developed for the Square-knot structure. Figure 1b illustrates the schematic diagrams and top views of all three fabricated origami-based sensors and the detailed fabrication technique and folding methods are presented in Figure S1a–c. The folding principles for the Love-knot and Pentagon-knot structures have been previously reported in our earlier work, and the same protocols were followed here. The Square-knot structure, introduced in this work, incorporates dual, triple, and quadruple resistive layers, optimizing sensitivity and accuracy by increasing the number of interfacial layers.

### 2.2. Characterization Methodologies

The morphological analysis of airlaid paper substrates with and without ink coatings was conducted using a tabletop scanning electron microscope (Hitachi T300, Hitachi High-Tech Corp., Tokyo, Japan) to examine the topography of the fabricated carbon ink films. The chemical properties of the composite ink were investigated by performing X-ray photoelectron spectroscopy (VG Scientific ESCALAB 250, Thermo Scientific, Waltham, MA, USA) and Raman spectroscopy (UniDRON microscopic Raman/PL spectroscopy, CL Technology Co., Ltd., New Taipei City, Taiwan). Their mechanical properties in elastic modulus were tested by using an MTS 42.503 Static Tensile Testing Machine (MTS Criterion 42.503 Test System, Eden Prairie, MN, USA). The electrical characterization of all tactile sensors was performed to observe the variation in electrical contact resistance (ECR) under applied pressure at room temperature. The sensors were affixed to a custom platform with strong adhesive tape to prevent multidirectional shear forces. Pressure was applied vertically using a JSV-500L stand with an ALGOL force gauge (ALGOL Instrument Co., Ltd., Taoyuan City, Taiwan). The ECR changes were monitored with a Keysight 34465A digital multimeter (Keysight Technologies, Inc., Santa Rosa, CA, USA).

The tactile sensors were further evaluated for wearable applications, including grasping, grip strength, and pulse-rate monitoring. The sensors were reconfigured as a wearable tactile sensing ring. For the grasping test, the ring was worn on the proximal phalanges of the middle finger to detect objects like a tennis ball and rubber bellow. In the grip strength test, it was placed on the intermediate phalanges, while for pulse-rate monitoring, the ring was worn on the distal phalanx of the right thumb. The output from the ring was connected to a MP36 physiological signal processing system (Biopac, Goleta, CA, USA). Grip strength was simultaneously measured using a dynamometer (SSL25LB, Biopac, Goleta, CA, USA), and pulse waveform was compared to electrocardiogram (ECG) signals recorded via a non-invasive cardiac output module (SS2L, Biopac, Goleta, CA, USA). All wearable tests were approved by the Internal Review Board (IRB) at the Research Ethics Center of National Taiwan University, with informed consent obtained from each participant.

## 3. Results

### 3.1. Sensing Mechanism

The sensing mechanism utilizes electrical contact resistance (ECR) variation, which was successfully employed in tactile sensing application by our group in our previous work [22,23,24,44,45,46]. This approach was inspired by ECR principles from Polymer Electrolyte Membrane (PEM) fuel cell research [47]. Electrical contact resistance (ECR) is a phenomenon that describes the resistance occurring between two conductive surfaces. When an external force or pressure is applied, the interface between these surfaces forms additional contact points, reducing overall resistance and allowing current to flow more easily (illustrated in Figure 1c). The conductive polyester-based graphene ink coated surface provides the ideal scenario for ECR variation. The charge carrier can pass through the conductive interfaces. By monitoring the variations in contact resistance, tactile forces or pressures can be effectively measured. Increased pressure results in more contact points, decreasing resistance and enhancing sensitivity. The textured surface roughness at the interface plays a critical role in maximizing ECR variation, improving sensor performance and response precision under applied pressure. This optimized design enables accurate pressure detection for a range of tactile sensing applications.

### 3.2. Material Analysis

**Figure 2:**
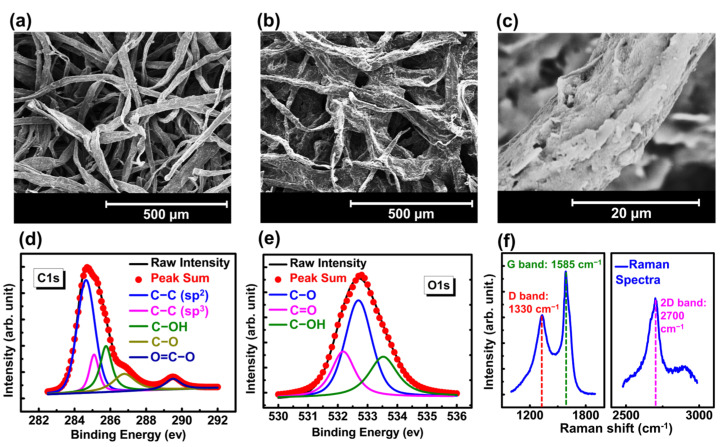
Material properties data: (**a**) SEM image of uncoated airlaid paper. (**b**) SEM image of screen-printed airlaid paper. (**c**) The magnified view of a single fiber, which shows the infusion of conductive ink into the fibrous structure of the substrate. (**d**) C1s and (**e**) O1s peaks of XPS spectra of conductive composite films. The deconvolution of these peaks were done to understand the bonds present in the material. (**f**) Raman spectra of conductive composite films showing the material composition of both inks. The D, G and 2D bands were explored to understand the presence of graphene layers.

The material analysis of the screen printed conductive airlaid papers are conducted and presented in Figure 2 to understand the properties of the sensing materials and its impact over device performance. In this work, we have used a commercially available graphene ink with the viscosity of 682.888 Pa·s at 1 s^−1^. The coating of this ink over fibrous airlaid paper was first monitored by the scanning electron microscopy method. Figure 2a shows the SEM image of a pristine airlaid paper with the randomly distributed cellulose fibers, which incorporates into the enhanced surface roughness. The topographic view of conductive airlaid paper with graphene ink was also demonstrated in the scanning electron microscope (SEM) image (Figure 2b). As observed, the ink was successfully infused into the paper and mimics the surface properties. The magnified SEM image of a conductive airlaid paper further confirms the phenomenon as we can see the graphene ink can successfully coat the cellulose fiber, which eventually incorporates into the overall surface roughness (Figure 2c). The fibrous roughness of the airlaid paper, as observed in SEM images, enhances the electrical contact points under pressure which is primarily resulted by the successful coating of the graphene ink.

X-ray photoelectron spectroscopy (XPS) and Raman spectra were measured to determine the surface chemical composition of graphene ink coated airlaid paper. High-resolution peak areas, line shapes, and intensities were obtained after the deconvolution of C1s and O1s peaks and are presented in Figure 2d,e. The position of the binding energy scale was adjusted to set the main C1s feature (C−C) at 284.6 eV, which is also the main peak as shown in Figure 2d [48]. Apart from that, the C1s spectrum also consists of several sub-peaks, which are resulted by the presence of both polyester binder and graphene-based conductive filler. In C1s spectrum, four other components are present at binding energies of 285, 285.8, 286.8 and 289.5 eV, denoting C−C (sp^3^), C−OH, C−O and O=C-O bonds, respectively [49,50,51,52]. The defects in graphene exist in the form of sp^3^ carbons, which can be found at 285 eV. The C-OH group at 285.8 eV shows the presence of reduced Graphene Oxide (rGO). The C-O bond at 286.8 can be attributed to the ester group of polyester and also the epoxy group. This epoxy functional group belongs to the rGO, which could be the primary component of the conductive filler. However, one distinct peak of O=C-O can be observed at 289.5 eV, clearly indicating the presence of polyester binder. On the other hand, the deconvolution of O1s spectrum shows the presence of C-O, C=O and C-OH bonds at 532.7, 532.2 and 533.5 eV respectively [53,54,55]. Among them, the C-O appears as the main peak which belongs to the polyester binder. The C=O peak present at 532.2 predominantly belongs to the rGO, however, it can also be influenced by polyester. Meanwhile, the C-OH bond present at 533.5 eV solidifies the presence of rGO as the graphene-based conductive filler in the ink.

This finding was further supported by the Raman spectra as shown in Figure 2f. Two main peaks were observed for the screen-printed airlaid papers. The D band appeared at 1330 cm^−^¹, associated with sp^3^-hybridized carbon atoms in a disordered state [56], while a more intense G band was present at 1585 cm^−^¹, corresponding to sp^2^-hybridized carbon atoms. The sharper feature of G band was attributed to sp^2^ phonon vibrations, confirming the presence of graphene in the polyester binder [57,58,59]. Moreover, a 2D band at 2700 cm^−^¹ indicates multiple layers of graphene [59]. The broad feature suggests that the graphene ink film contains a few layers with some defects. Furthermore, we have calculated the ratio between ID (intensity of D band) to the IG (intensity of G band). The reported ID/IG ratio of 0.81, being below 1, suggests a high degree of graphitization and contributes to enhanced electrical conductivity [59,60,61,62]. Therefore, it can be concluded that, with excellent surface mimicking ability and better conductivity, the reported polyester based graphene composite ink is a very good choice for tactile sensing devices.

### 3.3. Structural Analysis

**Figure 3:**
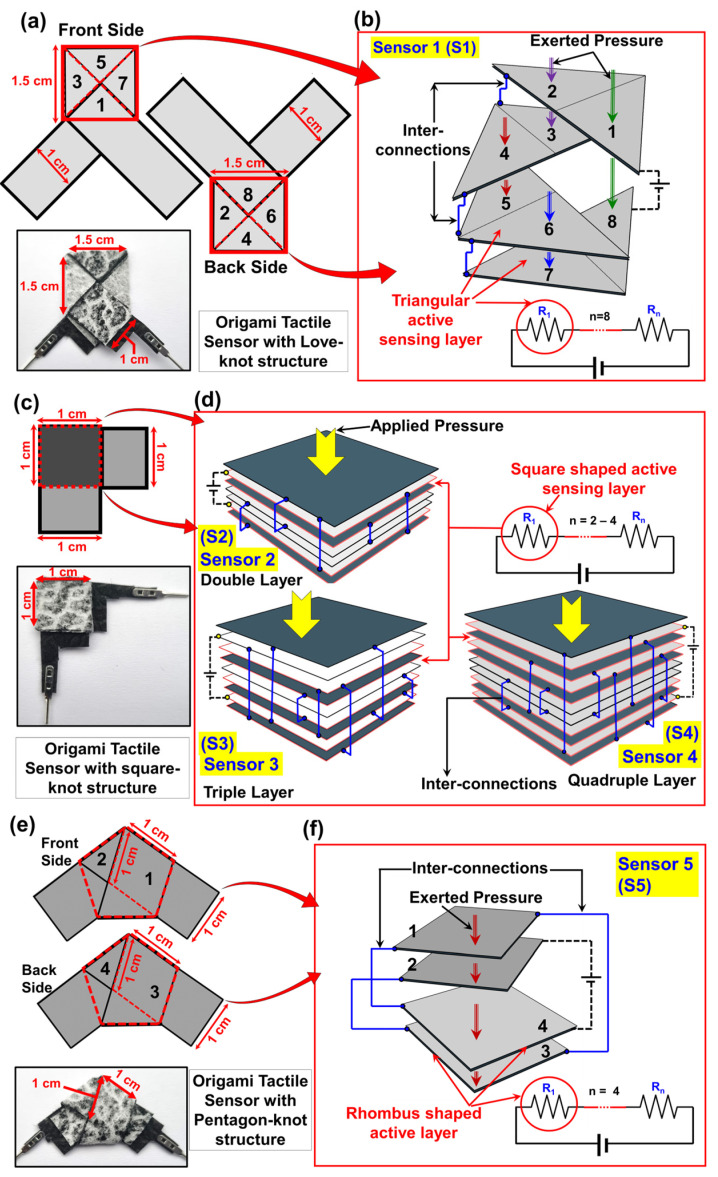
Circuit representation of Origami Tactile sensor. (**a**) The front and back side schematic of Love-knot origami tactile sensor (S1) showing the symmetrical arrangement of sensing layers. The real-time image of sensing device is also presented here. (**b**) The circuit representation of Love-knot structure. (**c**) The schematic diagram of Square-knot origami tactile sensor along with the real-time photograph of the device. (**d**) The circuit representation of Square-knot structure for three different configurations based on interfacial resistive layers’ numbers i.e., S2, S3 and S4. To identify those layers, the face-to-face arranged conductive papers were marked red borderline in this schematic. (**e**) The front and back side schematic of Pentagon-knot origami tactile sensor (S5), which also demonstrates the symmetrical arrangement of sensing layers. The real-time image of sensing device is also presented here. (**f**) The circuit representation of Pentagon-knot structure.

Analyzing the origami structures from an electrical circuit perspective is crucial for understanding the effect of ECR variation in tactile sensors. Figure 3a shows the schematic of the Love-knot origami tactile sensor (S1) alongside a real-time photograph. Each sensing region forms an isosceles right triangle, as seen from Supplementary Figure S1, resulting in eight triangular regions. The sensing area per triangle is 0.5 cm^2^, yielding a total sensing area of 4 cm^2^. Figure 3b presents the circuit model of S1, where each triangular interface (regions 1–8) acts as a resistor connected in series. Therefore, the total ECR at zero pressure can be interpreted in this way:

(1) $$R_{T}=\sum_{n=1}^{X}R_{n}$$

where R_T_ is the total ECR value and R_n_ is the ECR value for n number of interfacial layers (n = 0−X). The value of X changes for different origami structures, for example in this case the number X is equal to 8. Upon applying pressure, resistance decreases as force is transmitted across adjacent layers, causing changes in each sensing region, as indicated in the schematic. The overall thickness of the Love-knot structure is reported as 1505 µm.

An analogous evaluation was conducted for the Square-knot and Pentagon-knot origami tactile sensors too. Figure 3c presents the schematic of the Square-knot sensor, alongside a real-time photograph. The width of the paper strip was maintained at 1 cm, yielding a square sensing area of 1 cm^2^. The folding process, detailed in Supplementary Figure S1, illustrates that the structure is constructed by overlapping paper strips into multiple loops. By modulating the number of loops, the active sensing interfaces were systematically adjusted (Figure 3d). Three configurations—double, triple, and quadruple interfacial layers—were termed S2, S3, and S4, with thicknesses of 2610 µm, 3060 µm, and 3600 µm, respectively. The circuit representation demonstrates that with an increasing number of interfacial layers, the initial resistance escalates due to the series-connected resistors. Consequently, sensors with more interfacial layers exhibit greater ECR variation, leading to enhanced sensitivity and better tactile response.

Figure 3e illustrates the schematic diagram of the pentagon-knot origami structure (S5), along with its real-time photograph. Notably, the front and back sides of the structure are symmetrical, which can be attributed to its folding technique, detailed in Supplementary Figure S1. The rhombus-shaped sensing layers are formed with creases along the center and edges, designated as regions 1 and 3, respectively. Odd-numbered sensing regions overlap even-numbered regions, as shown in Figure 3f. The total thickness of S5 is reported to be 2670 µm. The circuit representation of S5 features four resistors connected in series at the interfacial layers, leading to an initial resistance of 970 kΩ. This higher initial resistance results from the larger interfacial gaps produced during folding. Unlike the compact structures of S2–S4, the S5 design allows greater deformation, which is expected to enhance the ECR variation.

The circuit representation also shows that these multiple layers can be formed in a limited footprint, while enhancing the interfacial resistive layers by causing multiple folds. Furthermore, the elastic modulus of the origami structure was also calculated by performing the compressive stress-strain tests. All origami samples were subjected to the compressive test by using a 5 N load cell to limit the maximum force to 5 N/m^2^. The average calculated elastic modulus, which can be expressed as the ratio between compressive stress and strain, are listed in Table 1.

It can be observed that the elastic modulus gradually increases for origami structure with higher number of interfacial layers. The highest recorded elastic modulus belongs to the Square-knot 3 structure with 455 kPa, which lies in the range of the elastic modulus of skin [63]. Therefore, a clear correlation between origami folding layers, thickness, and mechanical properties can be established here. All the parameters of three origami structures are presented in Table 1.

### 3.4. Resistive Sensing Characterization

**Figure 4:**
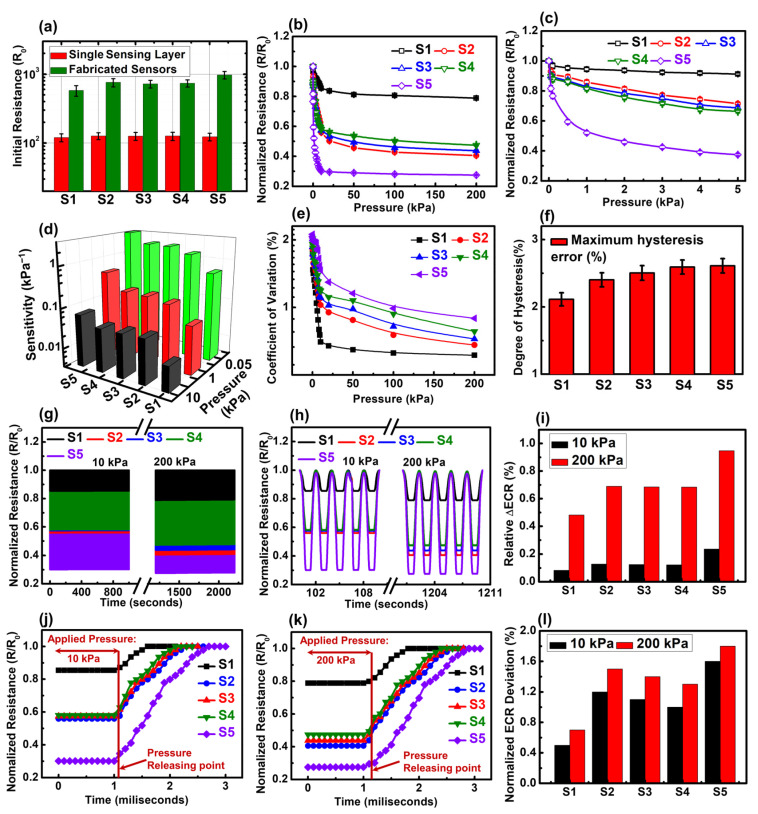
Resistive sensing characteristics of Origami tactile sensors. (**a**) The comparison of recorded initial resistance (R_0_) value of all five origami sensor configuration along with the R_0_ value of single sensing layer. Normalized resistance (R/R_0_) changes with applied pressure for all the origami tactile sensors for (**b**) the entire pressure range of 0–200 kPa and (**c**) low pressure region i.e., 0–5 kPa. (**d**) Sensitivity data of all five different fabricated sensors for three different pressures. (**e**) The co-efficient of variation data of all fabricated Origami tactile sensors indicating the stability and repeatability of sensor measurements. (**f**) The degree of hysteresis of all fabricated Origami tactile sensors indicating the accuracy of fabricated sensors. (**g**) Reversible testing for 2000 cycles of repeated loading and unloading of medium (10 kPa) and high (200 kPa) applied pressures. (**h**) Five number of cycles from medium- and high-pressure regions. (**i**) The relative ECR values of all fabricated origami sensors at 10 and 200 kPa to understand the resistance deviation in the end of loading-unloading cycle. The time-dependent resistance characteristics for tactile sensors with (**j**) 10 kPa and (**k**) 200 kPa applies pressure for one cycle. (**l**) The Recovery time of all five different sensors calculate from the time-dependent resistive data for one cycle.

The sensing characteristics of all multilayered Origami tactile sensors using graphene ink are presented in Figure 4. Measurements were conducted at room temperature across six samples to assess changes in normalized electrical contact resistance (ECR) under varying pressures. Normalized ECR is defined as the ratio of ECR at a specific pressure to that at zero pressure (R/R_0_). To provide a comprehensive comparison, the initial resistance values for single-layer and multilayer configurations are shown in Figure 4a. The initial resistance for single layers is relatively uniform, but increases with the number of interfacial layers in the multilayered sensors, as expected. For example, the initial resistance progressively increases from device S1 to S4, corresponding to the additional interfacial resistive layers, as previously discussed in Figure 3. Notably, device S5, with the Pentagon-knot fold, exhibits the highest initial resistance, attributed to both its increased layer count and its more loosely compacted structure. This suggests that S5 should display the most significant ECR variation, a hypothesis confirmed in subsequent sections by the normalized resistance variation data.

The resistive-sensing characteristics of the origami tactile sensors, based on five different folded configurations, are depicted in Figure 4b. Characterization was conducted at room temperature to examine the changes in normalized electrical contact resistance (ECR) under applied pressure. The normalized ECR is defined as the ratio of ECR at a given pressure to the ECR at zero pressure (R/R_0_). Five different sensor assemblies were prepared, and for each assembly, a minimum of six devices were measured. The applied pressure was capped at 200 kPa for this experiment. As shown in Figure 4b, all five fabricated sensors exhibit stable resistive characteristics. Two distinct sensing regions can be observed: a lower pressure region (0–10 kPa) with a sharp slope, indicating higher sensitivity, and a higher pressure region (10–200 kPa) with a flattened curve, signifying maximum electrical contact point generation. While this trend is consistent across the sensors, the response patterns vary. Device S1, with the Love-knot origami structure, shows the smallest ECR variation due to its compact assembly and lower initial resistance. Its dual-layer design limits scope of deformation. In contrast, device S5, featuring a Pentagon-knot structure, exhibits the largest ECR variation over both pressure regions, attributed to its four interfacial resistive layers and looser configuration, resulting in a higher initial resistance and more room for deformation.

For the Square-knot structures (S2, S3, S4), an interesting response pattern emerges when examining the relationship between the number of interfacial layers and their performance across different pressure ranges. At lower pressures (0–10 kPa), sensors with a higher number of interfacial layers, like S4, exhibited more pronounced ECR variation, likely due to the increased initial resistance and the cumulative effect of multiple resistive layers. This is further supported by the low-pressure ECR data (Figure 4c), which shows a steeper response for S4 compared to other devices. This behavior can be attributed to the increased number of interfacial contact points, which allows for greater electrical contact point generation at low pressures, resulting in higher resistance changes. As the number of interfacial layer increases, the device becomes more sensitive to subtle pressure changes in the low-pressure region. On the other hand, at higher pressures (10–200 kPa), the sensors with fewer interfacial layers, such as S2, showed better performance. This is predominantly due to the stacked structure characteristic of the Square-knot fold, where the interfacial resistive layers are separated by several conductive paper layers. This stacking leads to a more compact and rigid structure in devices with more layers (S3 and S4), reducing their capacity for deformation under higher pressures. As a result, the S2 device, with fewer layers and thus less structural rigidity, exhibited a greater capacity to respond to pressure in the higher range. As the number of stacked layers increases, the overall flexibility of the sensor decreases, with S4 having 11 stacked layers, S3 having 9, and S2 having 7. This increasing rigidity leads to more limited deformation at higher pressures for devices with more layers. Thus, while S4 and S3 exhibit greater sensitivity at lower pressures, S2’s simpler structure allows for more effective pressure sensing in the higher-pressure range.

The sensitivity of tactile sensors was further calculated from the resistance-pressure data with the following equation:

(2) S=∆RR0∆P

where S is the sensitivity, ΔR is the ECR difference, R_0_ is the initial resistance at zero pressure and ΔP is the pressure difference. The sensitivity data for all five sensors are presented in Figure 4d. Sensitivity increases significantly with a higher number of resistive layers, with the highest sensitivity observed at low pressures (0.05 kPa), making these sensors ideal for wearable applications. Device S5, with its Pentagon-knot structure and four interfacial resistive layers, shows the highest sensitivity, reaching 3.8 kPa^−1^ at 0.05 kPa. This highlights S5’s superior performance in low to medium pressure regions. The coefficient of variation (CV) for the fabricated sensors is shown in Figure 4e. CV, defined as the ratio of the standard deviation to the mean value of normalized ECR, indicates the stability and repeatability of the multilayered Origami tactile sensors. Measurements from over 10 samples reveal that sensors with fewer interfacial layers (S1) had lower CVs, while those with more layers, like S5, exhibited higher CVs due to the loose knot structure. Additionally, CV was significantly higher at lower pressures (0–1 kPa), indicating greater data variation at low pressures, likely due to fewer conduction points generated at the sensor interface compared to higher pressures.

The hysteresis behavior of the sensors was evaluated through 10 repeated pressure loading and unloading cycles, and the Degree of Hysteresis (DH) was calculated over the 200 kPa range. Hysteresis in tactile sensors refers to the difference or lag between the response during loading and unloading cycles at the same pressure point. It indicates the sensor’s accuracy and consistency, where lower hysteresis suggests better precision. This lag occurs due to the sensor material’s inherent memory effect, where deformation and recovery do not follow the exact same path, leading to discrepancies between the loading and unloading measurements. Supplementary Figure S2 shows the mean ECR variation for all five sensors during these loading and unloading cycles. DH is defined as the relative difference in the area under the normalized ECR-pressure curves for loading and unloading. The equation for DH is as follows [64,65]:

(3) $$DH=\frac{A_{unloading}-A_{loading}}{A_{loading}}\times 100\%$$

where A_Loading_ and A_Unloading_ are the area of the curves under loading and unloading, respectively. A relatively low hysteresis has been recorded for all the fabricated tactile sensors. Figure 4f illustrates that DH increases with more interfacial layers, with S1 having the lowest DH at 2.1%, due to its compact dual-layer design, while S5 exhibited the highest DH at 2.6%. This indicates that sensors with more layers, while offering higher sensitivity, are more prone to inaccuracies and deviations, as reflected by the higher CV and DH values.

Dynamic reversible testing was conducted on Origami Tactile sensors to evaluate repeatability and durability under medium (10 kPa) and high pressure (200 kPa). Figure 4g illustrates the reversible testing results for all five devices, with continuous loading/unloading at 10 and 200 kPa for 2000 cycles. The sensors demonstrated a stable dynamic response throughout the testing period. Additionally, to highlight the sensors’ cyclic performance, 5 consecutive cycles from both medium and high-pressure ranges are shown in Figure 4h, further confirming their consistent behavior under repeated pressure conditions. The overall resistance increases at the end of the 1000 cycles due to internal fatigue caused by long-term pressure loading. This was further confirmed by calculating the relative ECR deviation (ΔECR) by this equation:

(4) $$Relative∆ECR=\frac{{NECR}_{1st50}-{NECR}_{last50}}{{NECR}_{1st50}}\times 100\%$$

Here, NECR_1st 50_ stands for the normalized ECR for first 50 cycles, with the normalized ECR for last 50 cycles of the 1000 cycles termed as NECR _last 50_. The relative change in ECR percentage is shown in Figure 4i. The highest relative change in ECR was observed for S5 due to its loosely knotted structure, while devices with a more rigid configuration showed a lower relative change in ECR value. This still demonstrates good stability and durability. The normalized resistance data of one cycle were further analyzed after releasing both medium and low pressure to determine the recovery time of all fabricated devices as shown in Figure 4j,k respectively. It is evident that device S5 took longer to reach to its high resistance state owing to higher deformation. However, the recovery time decreases gradually for S2, S3, and S4 due to the increasing number of conductive papers, leading to higher rigidity and less deformation. The recovery time has been plotted in Figure 4l. It was observed that the Device S5 demonstrated the longest recovery time of 1.7 ms, which was recorded for Device 5 at 200 kPa. These data show that to attain quicker response and improved sensitivity, a careful folding optimization is essential. Based on the optimization, we have selected S2 and S5 for further applications.

### 3.5. Self-Packaging of Tactile Sensors by Origami Ring Formation

The origami structured tactile sensors were further compared to the planner tactile sensing devices with same number of interfacial layers. Supplementary Figure S3a,b show the sensing device structure with planner formation with and without packaging, where two conductive structural composite papers were face-to-face combined to form the resistive interfacial layers. The number of layers were further increased by simple folding method, which was introduced in our previous work [24]. The conductive paper was formed by following the same method introduced in this work, with the substrate and sensing materials kept the same (airlaid paper and graphene inks).

**Figure 5:**
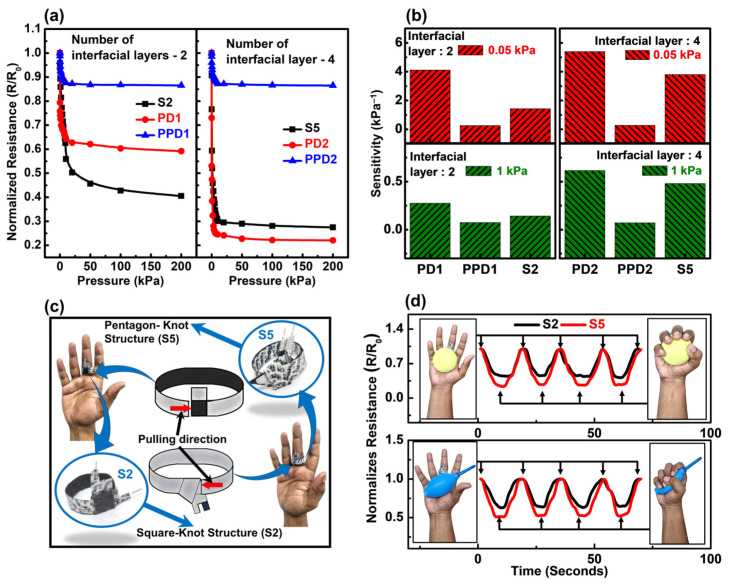
Comparison between origami structured tactile sensing device and planner sensing devices. (**a**) The resistive characteristics data of planner and origami tactile sensors for dual interfacial layers and quadruple interfacial layers. (**b**) The sensitivity comparison for 0.05 kPa and 1 kPa applied pressure. (**c**) The Formation method of Origami tactile sensing ring. It was created by using the extended paper strip to form a loop around the finger. Two sensors (S2 and S5) were selected as shown in the inset. The placement of origami ring was also demonstrated here. (**d**) Demonstration of wearable application by performing the grasping test by using two different objects, i.e., Tennis Ball and Rubber Bellow respectively.

The packaging was done by using an adhesive laminating film and presented and explained in Figure S3c. The planner device with two layers (PD1) and four layers (PD2) were formed by folding the paper only one time. After that packaging was done to form the packaged planner device (PPD1 and PPD2) for electrical characterization. Both PD1 and PPD1 were compared to S2 (Figure 3d) with two resistive interfacial layers as shown in Figure 5a. It was observed that S2 showcased relatively lower ECR value compared to PD1 at high pressure. However, at lower pressure the planner devices (PD1) have higher ECR variation as shown in Supplementary Figure S4. This is primarily due to the loose interfacial assembly for planner devices.

On the other hand, S5 (Figure 3f) with four interfacial layers showcased little higher ECR value compared to PD2 at both low and high pressure region as shown in both Figure 5a and Su. This is primarily due to the larger overall sensing area of PD2 since S5 possesses the rhombus shaped sensing region. However, the packaging process significantly reduces the performance of planner devices (PPD2) as shown in Figure 5a and Figure S4. The interfacial layers were tightly bound because of the strong airtight packaging process, thus resulting in very low room of deformation and compression. Therefore, we observe a very low ECR variation, leading to the lower sensitivity.

This phenomenon was further confirmed by sensitivity data of all six devices. The sensitivity values at 0.05 and 1 kPa applied pressures were presented in Figure 5b. The packaging causes a significant drop of sensitivity for both dual and quadruple layered tactile sensors. Meanwhile, the origami structured sensors have reasonably high sensitivity without using any laminating material for packaging. Figure 5b shows that at 0.05 and 1 kPa applied pressure sensitivity of origami structured devices are higher compared to the packaged devices. Therefore, the origami technique enables the self-packaged sensor assembly without significantly affecting the sensitivity value, making it suitable for wearable applications compared to the conventional planner devices. No plastic based lamination materials are needed in this process, which provides a sustainable and eco-friendly alternative for device assembly and packaging.

Thus, we have demonstrated the self-packaging functionality of the origami tactile sensors by transforming them into an adjustable ring using the same origami loop technique. One side of the sensor is intentionally extended and pulled through the outer layer to form a loop, resulting in an ultra-low-cost, adaptable origami ring for wearable applications. The dual-layer Square-knot (S2) and Pentagon-knot (S5) structures were selected due to their high sensitivity across low and high-pressure regions, and their ability to reconfigure into a self-packaged ring shape. In recent years, several studies were reported with regards to tactile sensing rings and their applications [66,67]. Compared to those studies, ours offer a simpler, more robust design, suitable for both vital sign monitoring and physical activity tracking. Figure 5c shows the self-packaged ring placed on the proximal phalanges region of middle finger, along with its real-time photograph. The initial assessment of the origami ring was conducted using a grasping test. The ring was worn on the middle finger, with two objects having different flexibility grasped. As shown in Figure 5d, a tennis ball was first selected for the test, with both S2 and S5 displaying stable, repeatable responses. The normalized ECR dropped when the ball was tightly grasped and increased during the resting phase. The same test was performed using a hollow rubber bellow (dust blower). During grasping, the bellow was squeezed, offering less resistance, leading to lower ECR variation compared to the tennis ball. This result indicates that the origami ring can effectively detect the structural properties of objects.

### 3.6. Demonstration of Wearable Sensing Application with Origami Ring

**Figure 6:**
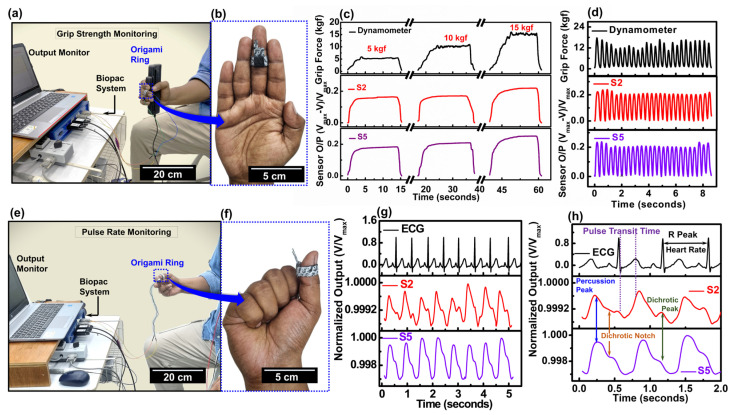
Grip Strength Monitoring. (**a**) Experimental set up of grip strength monitoring. (**b)** The close-up image of hand to show the origami ring location. (**c**) Isometric results of dynamometer and origami ring with S2 and S5 for three different applied force. (**d**) Isotonic test results of dynamometer and origami ring with S2 and S5 at rapid press and release movement. (**e**) Experimental set up of pulse rate monitoring. (**f**) The close-up image of hand to show the origami ring location. (**g**) Pulse-rate data detected by S2 and S5 origami ring compared to a standard ECG signal obtained simultaneously. (**h**) The three peaks of both pulse rate and ECG signal were emphasized for analyzing the pulse transit time (PTT).

The origami ring was further tested wearable sensing applications. Two separate tests were carried out, which includes grip strength and pulse-rate monitoring. The experimental setup for grip strength monitoring is shown in Figure 6a. The tactile sensing ring was adjusted and worn on the middle finger of the candidate’s right hand. A close-up image in Figure 6b shows its placement, ensuring that the sensing region remained fixed over the intermediate phalanges of the middle finger. This positioning was further clarified in the anatomical schematic diagram presented in Supplementary Figure S5a. A hand dynamometer (SSL25LB, Biopac, Goleta, CA, USA) was used to apply forces of 5, 10, and 15 kgf, while both the sensor and dynamometer outputs were collected using a Biopac physiological signaling processing system. The relative output voltage of the origami ring was calculated using the equation

(5) $$Sensor\;Output=\frac{V_{max}-V}{V_{max}}$$

V_max_ is the maximum voltage while V is the instantaneous voltage. The results presented in Figure 6c show the isometric or static responses of dynamometer and origami ring. To conduct isometric tests, the dynamometer was held for a certain period of time (15–30 s) to exert the desired force and to conduct the isometric test. The results show a significant response from both S2 and S5 origami ring. The origami ring with S5 demonstrated the better result compared to S2 owing to the multilayered structure as discussed previously since S5 showcases higher sensitivity. The rise-time for dynamometer and two origami rings were further calculated and presented in Supplementary Figure S6a. The rise time is defined as the time required to reach the peak value of applied force or sensor output. The results show that the rise-time for 15 kgf exerted force is much higher (10.71 s) compared to 10 kgf (8.838 s). Interestingly, while the dynamometer took longer to reach peak force, the origami sensors showed relatively faster response, likely due more electrical contact point generation at higher pressures as we have previously discussed.

This was further confirmed by the steady state linearity, which was calculated using linear fitting from the output curve’s steady state region for both the dynamometer and origami ring at three different pressures. This region corresponds to when the candidate maintained a constant force. The Supplementary Figure S6b shows that the dynamometer has higher steady state linearity, suggesting possible fluctuations in output due to the candidate’s grip strength and conditioning. In contrast, S2 and S5 demonstrate lower linearity, indicating better stability at higher pressures but also reaching saturation. Therefore, further optimization of the origami tactile sensing ring is needed to improve accuracy in real-time high-pressure situations. Lastly, we have also performed the isotonic testing. The candidate was asked to perform rapid grip-and-release movements. Figure 6d shows the obtained data and for rapid isotonic testing the origami rings demonstrate output signal at the exact same time and there are no lags in the rise time for dynamic movements. These results show that the origami rings have potential to be used for future therapeutic applications.

The origami sensing ring was effectively tested for real-time pulse detection, as shown in Figure 6e. Worn on the distal phalanx of the right thumb, the sensor was connected to the Biopac physiological signaling processing system for monitoring (Figure 6f). The anatomical schematic in Supplementary Figure S5b shows the ring positioned over the princeps pollicis artery, responsible for perfusing the thumb’s bones, muscles, and skin. Pulse data from both S2 and S5 rings were compared to simultaneous ECG measurements, with both rings securely worn for accurate pulse rate sensing. Figure 6g illustrates the pulse waves successfully detected by the S2 and S5 rings, compared with the ECG signal. A section featuring three peaks was analyzed further, as shown in Figure 6h, clearly displaying the percussion peak, dichrotic peak, and dichrotic notch in the pulse waveform. In Figure 6h we observed a lag between the ECG and pulse wave. This delay, known as pulse transit time (PTT), results from the time it takes for the blood to travel from the heart to the point of measurement in the body [68,69,70]. For convenience, it is usually measured from the R wave on the electrocardiogram to the characteristic p-base point or virtual base point of the pulse signal [70]. The calculation of PTT was also carried out in similar way as depicted in Figure 6h. The recorded PTT was at 259 ms highlighting the potential of the origami sensing ring for cardiovascular monitoring.

## 4. Discussion

Although origami and folding techniques have recently been explored in paper-based tactile sensors—leveraging paper’s flexibility, compressibility, and biodegradability [36,37,38,39,40]—few studies have systematically evaluated or optimized these designs for device performance and specific applications, nor fully exploited the advantages offered by complex 3D origami structures. Our study broadens this scope by:

(1) Enhancing the structural stability and self-packaging of the sensors: Planar sensors often require encapsulation layers for protection in wearable applications, which can significantly degrade sensitivity. In contrast, our origami folding inherently integrates packaging into the structure. Knot-shaped designs form loop-based systems that secure face-to-face paper strips and shield the sensing layers within outer folds, enhancing durability without compromising performance.

(2) Demonstrating the robustness and compatibility of origami structures: The increased folds and interfacial layers result in an enhanced elastic modulus, yet it remains within the range of human skin, ensuring compatibility for wearable applications. These sensors are also low-maintenance and perform reliably under ambient conditions, making them suitable for extreme environments.

(3) Modeling the electrical behavior of various origami configurations to identify designs with enhanced sensitivity or faster response: By benchmarking against previous work (Table S1) and detailing our findings (Table 1), we show that the S5 Pentagon knot, with its relatively loose configuration, achieves higher sensitivity, but at the expense of accuracy. In contrast, the S2 Square knot offers greater precision and faster recovery times due to its simpler structure. These findings demonstrate how origami-based designs can be tailored to specific needs: S5 is ideal for high-sensitivity applications, whereas S2 provides a balance between sensitivity and precision, making it particularly suitable for wearable devices requiring stability and real-time feedback.

In addition, the selection of conductive ink was crucial to ensure consistent performance during origami folding. The polyester-graphene composite ink used in this study diffuses effectively into the fibrous structure of airlaid paper and remains crack-free even after multiple uses, outperforming carbon black-based electronic paints previously tested [23]. Material analysis revealed that the ink contains reduced graphene oxide (rGO), which contributes to higher initial resistance compared to pristine graphene. This higher initial resistance amplifies changes in ECR at the surface level, enhancing sensitivity and making the sensors particularly effective for subtle pressure detection. Moreover, by implementing the origami folding, the sensing area and the resistance value at the interface of graphene ink coated airlaid papers can be manipulated for better performance.

Our origami ring can detect both subtle and high-pressure stimuli, with applications such as grip strength monitoring and pulse rate detection. Handgrip strength is a reliable indicator of physical function and health status [71,72], and performing isometric resistance tests has been shown to lower resting blood pressure [73,74]. The ring provides a cost-effective alternative to traditional grip strength testers, allowing patients to wear the device and press any suitable material to perform isometric handgrip testing conveniently at home. Beyond healthcare, this functionality extends to fitness and sports, where athletes can monitor strength progression through isotonic and isometric exercises, making it a versatile tool for a range of applications.

For subtle pressure detection, pulse monitoring was performed directly on the thumb, offering a simpler, more flexible, and robust alternative to previously reported tactile sensors [75,76,77]. The subtle pressure generated by pulse waveform influenced origami sensing ring’s interfaces with the charge carrier conduction influenced at that time. The rough surfaces can cause higher ECR variation at subtle pressure as we have shown in our theoretical model, and the same phenomena can be observed during pulse waveform detection since ECR value goes down under the influence of pulse wave. The pulse waveform was successfully detected and analyzed alongside ECG signals to extract pulse transit time (PTT), an essential cardiovascular biomarker. Changes in PTT are linked to health conditions such as atherosclerosis, blood pressure dysregulation [78], reduced cardiac function, pulmonary hypertension, and heart failure [79,80]. By integrating pulse-rate detection with grip-strength functionality, the origami ring demonstrates its potential as a cost-effective, multifunctional tool for physiological monitoring and rehabilitation.

## 5. Conclusions

This work introduces a multi-layered origami tactile sensing ring with a sensing range of 0–200 kPa, ideal for wearable applications. The study explored five configurations using three distinct origami structures (Love-knot, Square-knot, and Pentagon-knot), coated with polyester-graphene conductive composite ink. The sensors were depicted in their circuit representations to further explain the resistive sensing behavior along with their multilayered structure. Circuit representations and multilayer designs highlight the resistive sensing behavior, driven by Electrical Contact Resistance (ECR) variations across face-to-face conductive paper layers. The folding technique enhances the number of interfacial regions within a compact footprint, achieving high sensitivity at 3.8 kPa^−1^ at 0.05 kPa, with room for further optimization in response speed and accuracy. Wearable applications, including grip-strength and pulse detection, demonstrate the ring’s effectiveness across pressure ranges. Positioned directly on the finger, the sensor capitalizes on arterial proximity for precise readings. The origami design leverages substrate bendability, tackling challenges in miniaturization while offering a cost-effective and integrable solution. This work advances flexible electronics and wearable technology, providing accessible, sustainable, and precise health monitoring suitable for physiological monitoring, cardiovascular diagnostics, and rehabilitation.

## Figures and Tables

**Table 1:** Parametric details of Origami Tactile Sensors presented in this work.

Origami Structure	Single Sensing Region Shape	Interfacial Overlapped Layers	Overall Thickness (µm)	Average Elastic Modulus (kPa)	Overall Resistance (kOhm)
Love-knot	Triangle	2 x 4 i.e., 4 dual layers	1505	~190	580
Square-knot	Square	2 layers separated by multiple papers	2610	~344	740.5
		3 layers separated by multiple papers	3060	~407	737.8
		4 layers separated by multiple papers	3600	~455	736.7
Pentagon-knot	Rhombus	4 layers arranged back-to-back	2670	~284	973.5

## Data Availability

Data sharing is not applicable to this article.

## Supplementary Materials

The following supporting information can be downloaded at: https://www.mdpi.com/article/10.3390/bios15010008/s1. Figure S1: The origami folding technique for (a) Love-knot origami structure, (b) Square-knot origami structure, and (c) Pentagon-knot origami structure. Figure S2: Hysteresis loops of sensors with (a) S1 (Love-knot structure), (b) S2 (dual layered Square-knot structure), (c) S3 (triple layered Square-knot structure), (d) S4 (quadruple layered Square-knot structure), and (e) S5 (Pentagon-knot structure). The hysteresis loop was presented here for the pressure range of (0–200 kPa); Figure S3: Schematic diagram of planner devices with (a) dual resistive layer; (b) quadruple resistive layers with and without packaging; and (c) The packaging method for planner devices. The sensor was placed in between two adhesive materials that were laminated; Figure S4: Comparison between origami structured tactile sensing device and planner sensing devices. The resistive characteristics data of planner and origami tactile sensors for (a) dual interfacial layers and (b) quadruple interfacial layers at lower pressure region i.e., 0–5 kPa; Figure S5: Placement of origami sensing ring: (a) The location of origami tactile sensing ring on intermediate phalanges of the middle finger for grip test monitoring. The sensor placement was at the tendon of flexor digitorum profundus. (b) The location of origami tactile sensing ring at the thumb. It was positioned over the princeps pollicis artery, responsible for perfusing the thumb’s bones, muscles, and skin; Figure S6: Extracted parameters to analyze the dynamometer and origami rings performance by plotting (a) rise time and (b) steady state linearity; Table S1: Comparison of previously reported paper based origami pressure/tactile sensors with the re-ported work.
