# Supplementary material for: A Multi-Layered Origami Tactile Sensory Ring for Wearable Biomechanical Monitoring
Source: Biosensors (Basel). 2024 Dec 27;15(1):8. doi: 10.3390/bios15010008 (PMC11763825; doi:10.3390/bios15010008)
Supplement: Supplementary file 1 [file biosensors-15-00008-s001.zip › biosensors-3365911-supplementary.pdf]

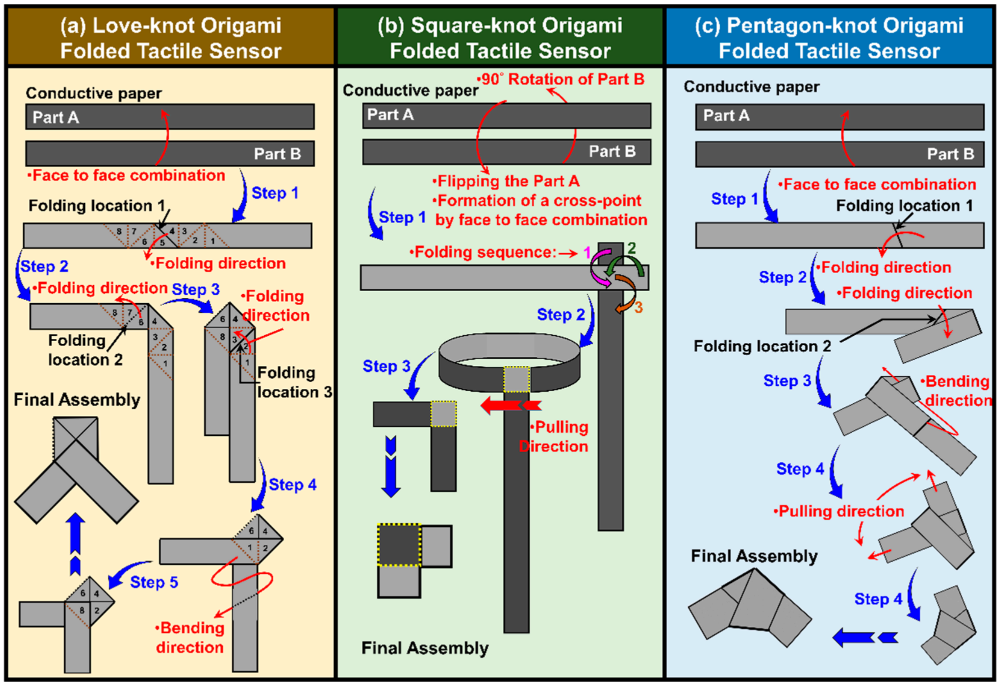

Figure S1: The origami folding technique for (a) Love-knot origami structure, (b) Square-knot origami structure, and (c) Pentagon-knot origami structure.

.

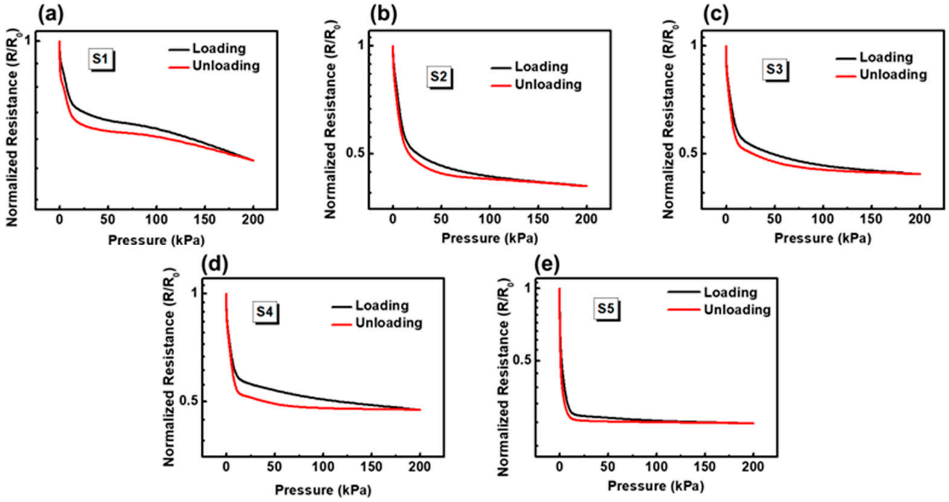

Figure S2: Hysteresis loops of sensors with (a) S1 (Love-knot structure), (b) S2 (dual layered Square-knot structure), (c) S3 (triple layered Square-knot structure), (d) S4 (quadruple layered Square-knot structure), and (e) S5 (Pentagon-knot structure). The hysteresis loop was presented here for the pressure range of (0-200 kPa).

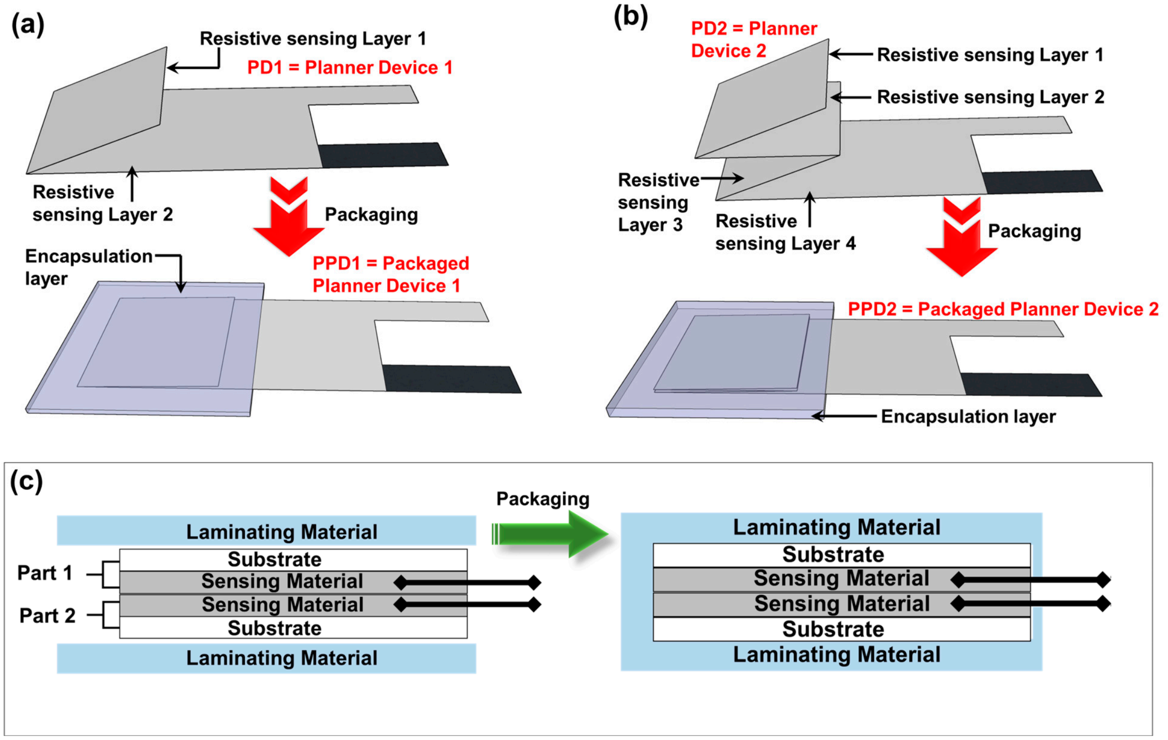

Figure S3: Schematic diagram of planner devices with (a) dual resistive layer; (b) quadruple resistive layers with and without packaging; and (c) The packaging method for planner devices. The sensor was placed in between two adhesive materials that were laminated.

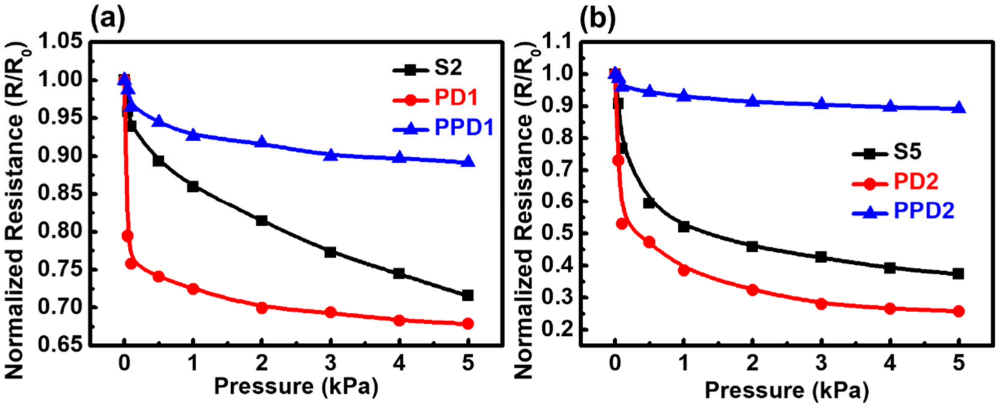

Figure S4: Comparison between origami structured tactile sensing device and planner sensing devices. The resistive characteristics data of planner and origami tactile sensors for (a) dual interfacial layers and (b) quadruple interfacial layers at lower pressure region i.e. 0-5 kPa.

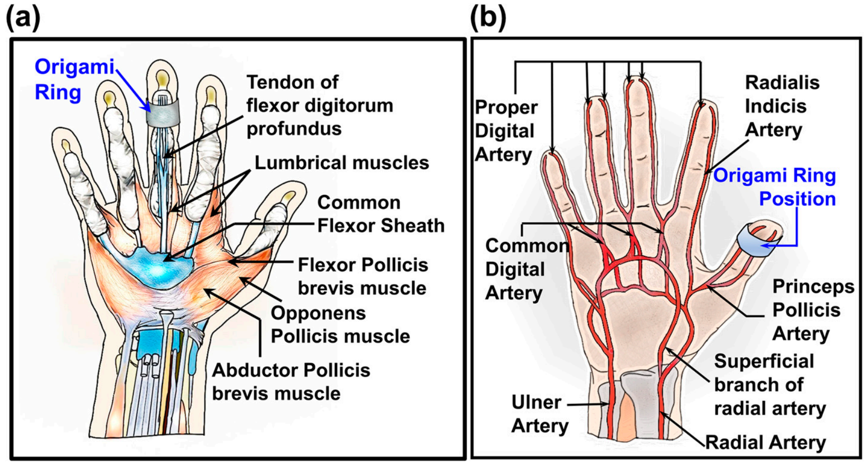

Figure S5 Placement of origami sensing ring: (a) The location of origami tactile sensing ring on intermediate phalanges of the middle finger for grip test monitoring. The sensor placement was at the tendon of flexor digitorum profundus. (b) The location of origami tactile sensing ring at the thumb. It was positioned over the princeps pollicis artery, responsible for perfusing the thumb's bones, muscles, and skin.

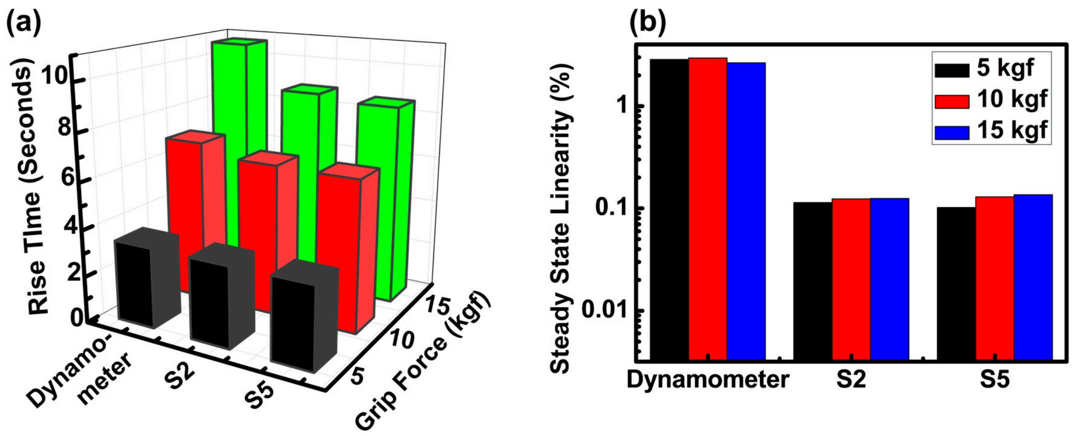

Figure S6 Extracted parameters to analyze the dynamometer and origami rings performance by plotting (a) rise time and (b) steady state linearity.

Supplementary Table S1. Comparison of previously reported paper based origami pressure/tactile sensors with the reported work.

Origami Structure	Measured Parameters	Sensitivity	Origami Structure Optimization
Stacking the conductive papers and patterns formed by cut.[1]	Resistance	2.36 kPa^{-1} at less than 30 kPa	N/A
Dual layer single fold, Paper crane, origami gripper.[2]	Capacitance	1.0 nF/(kPa cm2) below 200 kPa	N/A
Paper Crane, Fingertip folded sensor.[3]	Resistance	7.65 kPa^{-1} at 0–3.3 kPa	N/A
Combination of cylindrical origami papers.[4]	Capacitance	1.96 kPa^{-1} at 0−50 kPa	Different radii, structures, thicknesses, and distances.
Folded Structure inside an ecoflex shell.[5]	Capacitance	0.051 kPa^{-1} at less than 1 kPa	Overall Thickness Optimization
Three different knot shaped structure – Love-knot, Square knot and Pentagon Knot - (This work).	Resistance	3.8 kPa^{-1} at 0.05 kPa	Different structures based on their circuit representation

In this table the sensitivity values of the origami tactile sensors were compared. It can be observed that all the aforementioned works have implemented either capacitive or resistive structure. However, the structure based optimization was not really focused by others. In our work, we have compared three different origami based structures and carefully optimized to obtain the sensitivity at subtle pressure range (0.05 kPa) which is ideal for wearable applications.

References

1. Liu, X.; Sun, J.; Tong, Y.; Zhang, M.; Wang, X.; Guo, S.; Han, X.; Zhao, X.; Tang, Q.; Liu, Y. Calligraphy and Kirigami/Origami-Inspired All-Paper Touch–Temperature Sensor with Stimulus Discriminability. *ACS Appl. Mater. Interfaces.* **2023**, *15*, 1726–1735.
2. Li, S.; Chu, J.; Li, B.; Chang, Y.; Pan, T. Handwriting Iontronic Pressure Sensing Origami. *ACS Appl. Mater. Interfaces* **2019**, *11*, 46157–46164, doi:10.1021/acsami.9b16780.
3. Yao, D.-J.; Tang, Z.; Zhang, L.; Liu, Z.-G.; Sun, Q.-J.; Hu, S.-C.; Liu, Q.-X.; Tang, X.-G.; Ouyang, J. A Highly Sensitive, Foldable and Wearable Pressure Sensor Based on Mxene-Coated Airlaid Paper for Electronic Skin. *J. Mater. Chem. C.* **2021**, *9*, 12642–12649, doi:10.1039/D1TC02458B.
4. Guo, X.; Li, Y.; Hong, W.; Yan, Z.; Duan, Z.; Zhang, A.; Zhang, X.; Jin, C.; Liu, T.; Li, X.; et al. Bamboo-Inspired, Environmental Friendly PDMS/Plant Fiber Composites-Based Capacitive Flexible Pressure Sensors by Origami for Human–Machine Interaction. *ACS Sustain. Chem. Eng.* **2024**, *12*, 4835–4845, doi:10.1021/acssuschemeng.3c06949.
5. Li, J.; Godaba, H.; Zhu, J. Paper-Based Origami Transducer Capable of Both Sensing and Actuation. *Extrem. Mech. Lett.* **2021**, *49*, 101507.
